# Spatially divergent metabolic impact of experimental toxoplasmosis: immunological and microbial correlates

**DOI:** 10.1128/msystems.01126-25

**Published:** 2025-11-06

**Authors:** Mahbobeh Lesani, Caitlyn E. Middleton, Tzu-Yu Feng, Jan Carlos Urbán Arroyo, Eli Casarez, Sarah E. Ewald, Laura-Isobel McCall

**Affiliations:** 1 Department of Microbiology and Plant Biology, University of Oklahoma 6187, Norman, Oklahoma, USA; 2 Department of Chemistry and Biochemistry, San Diego State University 7117, San Diego, California, USA; 3 Department of Microbiology, Immunology and Cancer Biology at the Carter Immunology Center, University of Virginia School of Medicine 12349, Charlottesville, Virginia, USA; 4 Department of Chemistry and Biochemistry, University of Oklahoma 6187, Norman, Oklahoma, USA; University of Wisconsin-Madison, Madison, Wisconsin, USA

**Keywords:** *Toxoplasma gondii*, metabolism, mass spectrometry, inflammation, microbiome

## Abstract

Maladaptive host metabolic responses to infection are emerging as major determinants of infectious disease pathogenesis. However, the factors regulating these metabolic changes within tissues remain poorly understood. In this study, we used toxoplasmosis, as a prototypical example of a disease regulated by strong type I immune responses, to assess the relative roles of current local parasite burden, local tissue inflammation, and the microbiome in shaping local tissue metabolism during acute and chronic infections. Toxoplasmosis is a zoonotic disease caused by the parasite *Toxoplasma gondii*. This protozoan infects the small intestine and then disseminates broadly in the acute stage of infection, before establishing chronic infection in the skeletal muscle, cardiac muscle, and brain. We compared metabolism in 11 sampling sites in C57BL/6 mice during the acute and chronic stages of *T. gondii* infection. Strikingly, major spatial mismatches were observed between metabolic perturbation and local parasite burden at the time of sample collection for both disease stages. By contrast, a stronger association with indicators of active type I immune responses was observed, indicating a tighter relationship between metabolic perturbation and local immunity than with local parasite burden. Loss of signaling through the IL1 receptor in IL1R knockout mice was associated with reduced metabolic impact of infection. In addition, we observed significant changes in microbiota composition with infection and candidate microbial origins for multiple metabolites impacted by infection. These findings highlight the metabolic consequences of toxoplasmosis across different organs and potential regulators.

**IMPORTANCE:**

Inflammation is a major driver of tissue perturbation. However, the signals driving these changes on a tissue-intrinsic and molecular level are poorly understood. This study evaluated tissue-specific metabolic perturbations across 11 sampling sites following systemic murine infection with the parasite *Toxoplasma gondii*. Results revealed relationships between differential metabolite enrichment and variables, including inflammatory signals, pathogen burden, and commensal microbial communities. These data will inform hypotheses about the signals driving specific metabolic adaptation in acute and chronic protozoan infection, with broader implications for infection and inflammation in general.

## INTRODUCTION

Toxoplasmosis is a zoonotic disease caused by *Toxoplasma gondii* parasites, organisms that are estimated to infect more than 30% of the world population. Two billion people are infected by *T. gondii*. Latin America, Africa, Eastern/Central Europe, and Southeast Asia have the highest infection rate (1). Once infected, patients often harbor *T. gondii* for life. *T. gondii* infection is controlled by a canonical Type I CD8+ T-cell response (2). Immunocompetent patients have mild, flu-like symptoms that are often undiagnosed at the time of infection. Immune-compromised patients, including people with HIV with a low number of T cells and patients receiving immunosuppressive therapies for cancer or organ transplants, are susceptible to primary infection and to the recrudescence of chronic infection leading to *Toxoplasma* encephalitis and uncontrolled systemic infection if treatment is not provided (3). One recent study estimated that more than 13 million people living with HIV are co-infected with *T. gondii* worldwide (4). In immunocompetent mothers, primary infection during pregnancy can lead to congenital toxoplasmosis, causing miscarriage, stillbirth, or chorioretinitis, hydrocephalus, and intracranial calcifications (3). An estimated 190,000 new cases of congenital toxoplasmosis occur annually, in addition to the 1.2 million existing ones (5).

*T. gondii* has the remarkable ability to infect most nucleated cell types *in vitro* (6). *In vivo*, the parasite initially invades the small intestine, infecting stromal cells and infiltrating immune cells, which play dual roles, both mediating systemic infection and clearing parasites. IFN-γ plays a critical role in initiating immune clearance of *T. gondii* and polarizing the protective adaptive immune response, which peaks around 14 days after infection (7). The parasite has developed strategies to evade sterilizing immunity, converting from the rapidly dividing tachyzoite phenotype that dominates acute infection to slowly dividing bradyzoites, which form long-lived tissue cysts in the brain, cardiac, and skeletal muscle as well as in visceral organs, including the lungs, liver, and kidneys at lower frequency (8, 9).

After infection, the homeostatic balance of glycolysis, glutaminolysis, and fatty acid oxidation shifts to activate immune cells and provide substrates for defense mechanisms against pathogens (10, 11). There is a growing appreciation that metabolism is altered in acute and chronic *Toxoplasma* infection. Studies in BALB/c mice infected with *T. gondii* showed higher metabolic perturbation in the acute stage compared to the chronic stage in the liver (12), brain (13), and spleen (14). Liver and brain cholesterol levels were reduced during acute *T. gondii* infection in Swiss Webster mice but normalized during chronic infection (15). Chronic infection with *T. gondii* can result in the progressive muscle-wasting disease called cachexia (16–18). This has been associated with increases in some serum sphingolipid and glycerophospholipid species and decreased levels of a number of TCA cycle intermediates (19). Previous studies have shown that metabolic perturbation can differ between organs such as the brain (20), serum (19), spleen (14), and liver (12). These studies suggest that metabolic perturbations may depend on location (e.g., organ/tissue type) and time point (acute versus chronic stages of infection), parameters linked to shifts in parasite abundance and immunity. However, a study directly comparing these parameters has not previously been performed.

To understand the local drivers of host metabolic alterations during *T. gondii* infection, we assessed eleven organs during the acute (15 days post-infection) and chronic (50 days post-infection) stages of *T. gondii* infection. Significant metabolic impacts were observed in the liver, heart, duodenum, ileum, quadriceps, cecum, and intestinal contents during acute infection. By contrast, at the chronic time point, significant overall metabolic impact was only observed in the cecum, large intestine, and intestinal contents. This corresponded with sustained shifts in commensal microorganisms. We found the tightest association between metabolic perturbations and immune responses, whereas no clear spatial association with local tissue parasite burden at the time of metabolome sampling was observed. These results help us understand the microbial and inflammatory drivers of organ-specific metabolic responses to parasitic diseases over time. In the long term, this work will enable the identification of metabolic pathways that can be modulated to improve disease symptoms without compromising parasite clearance, consistent with a disease tolerance framework (21).

## MATERIALS AND METHODS

### Mice

C57BL/6 and IL-1R-deficient (B6.129S7-*Ilr1^tm1/mx^/*J, IL1RKO) male mice were purchased from the Jackson Laboratory. Mice were housed in the University of Virginia animal facility for 2 weeks prior to infection, and bedding was mixed to help homogenize the microbiome and minimize cage effects.

### Infections and food intake monitoring

Peroral infections were conducted with 25T. *gondii* cysts of the Type II Me49 strain stably expressing green fluorescent protein (GFP) and luciferase as previously described (22). Briefly, cysts were harvested from chronically infected CBA/J mice, mashed through a 50 micron sieve, and stained with *Dolicos biflorus* agglutinin conjugated to rhodamine (Vector Laboratories). Cysts were counted at 20× magnification based on GFP, rhodamine signal, and morphology. Experimental mice were fasted overnight and infected by pipetting into the mouth. Mice were housed on wood chip bedding. Mice were weighed daily and monitored for moribund behavior. Experiments were set up with groups of 5 mice per infection and repeated twice (10 total per condition). The experimental N value for uninfected groups is C57BL/6 WT *N* = 10 (15 days post-infection and 50 days post-infection); IL1RKO *N* = 10 (15 days post-infection). For acute 15 days post-infection C57BL/6 WT *N* = 9 because 1 mouse did not seroconvert (not infected); IL1RKO *N* = 10. For the chronic 50 day infection, C57BL/6 WT *N* = 7 due to animals meeting euthanasia criteria before the experimental endpoint.

### Tissue harvest for metabolomics

Mice were perfused intracardially with 10  mL phosphate-buffered saline. 30–50 mg of each tissue was harvested as follows. For the small intestine, cecum, and large intestine, contents were gently eliminated from the tissue with curved forceps and a sample of the slurry was isolated. The cecum, large intestine, duodenum, and the central portion of the jejunum and ileum segments were isolated. A segment of the right lobe of the liver, the apex, and ventricular region of the heart and a quadricep were collected. Samples were flash-frozen in liquid nitrogen using pre-weighed tubes and tissue mass was determined.

### Metabolite extraction

Metabolite extraction was performed as described in Want et al. (23). We have previously validated this method in a variety of infectious disease models (24–27), so that its implementation here enables cross-study comparison. Tissues were resuspended at 50 mg per 175 µL of chilled LC-MS-grade water (Fisher Optima), on ice. Tissues were homogenized by bead beating with a 5 mm steel ball in a pre-chilled TissueLyser (Qiagen) at 25 Hz for 3 min. A fraction of homogenate was set aside and frozen for DNA extraction. For aqueous metabolite extraction, LC-MS-grade methanol (Fisher Optima) spiked with 4 µM sulfachloropyridazine (Sigma-Aldrich) was added to 30 mg (by volume) of homogenized sample to a final concentration of 50% methanol. The sample was homogenized in a chilled TissueLyser (Qiagen) at 25 Hz for 3 min. The homogenate was centrifuged for 15 min at 14,000 × *g* at 4°C to eliminate debris. The supernatant was dried in a Savant SPD1030 SpeedVac (Thermo Fisher Scientific), sealed, and stored at −80°C. For organic metabolite extraction, the centrifugation pellet was resuspended in 3:1 (by volume) dichloromethane (Fisher Optima): methanol solvent mixture and further homogenized at 25 Hz for 5 min. Samples were centrifuged at 14,000 *× g* for 2 min at 4°C to eliminate debris. The second centrifuged supernatant was collected and air-dried in the fume hood, sealed, and stored at −80°C.

### Liquid chromatography-tandem mass spectrometry data acquisition

All LC-MS analysis methods were previously optimized for tissue small molecule characterization (26, 28). Organic and aqueous extractions were merged by resuspending in 150 µL of 50% MeOH spiked with 2 µM sulfadimethoxine and transferred to a new plate after sonication and centrifugation (5 min, 14,800 RPM). The samples were separated through a C8 LC column (Phenomenex; 0.7 µm, 50 mm × 2.1 mm, 100 Å Kinetex) with a C8 guard cartridge, followed by LC-MS/MS acquisition in positive mode (Thermo Scientific Vanquish UHPLC system and Q Exactive Plus MS). Instrument performance was verified by acquiring data from a standard mix of six known small molecules and monitored by assessing peak shape and retention time of the internal standards throughout data acquisition. The 2,994 highest abundance *m/z* found in blank samples and the 6 *m/z* from our standard mix of known molecules were added to the method as an exclusion list so that MS/MS spectra are not acquired from metabolite features found in the blank. Exclusion list size is capped at 3,000 entries by the instrument control software. Instrument methods were as in reference 29.

### Liquid chromatography-tandem mass spectrometry data analysis

Collected raw data were converted to mzXML with MSConvert software (version 3.0.19014-f9d5b8a3b, Proteowizard, Palo Alto, Santa Clara, CA, USA) (30). mzXML data were processed through MZmine 2 (version 2.53) to generate the feature table (31). All MZmine data analyses parameters are in Table S1. Due to observing a batch effect (from changing the column during the run due to high backpressure and changes in peak shape), WaveICA batch removal method was applied to normalize peak area (parameters: alpha = 1, cut-off = 0.1, K = 10). In comparative work, WaveICA outperformed all but one batch normalization method on a large data set (32). PCoA plots (Bray–Curtis dissimilarity metric), PERMANOVA, and distance analysis to detect differences between groups were done through QIIME2 (33, 34). To annotate the features and visualize annotation mirror plots, GNPS (Global Natural Products Social molecular networking) was used (35, 36), and annotations retrieved using an automated code (25). All annotations were visually inspected for match quality and for biological plausibility, as described in Theodoris et al. (37). All annotations are at confidence level 2/3, according to the metabolomics standards initiative (38). GNPS parameters were as in (39). MolNetEnhancer was used to classify features into chemical families (40) using ClassyFire chemical ontology (41). To visualize the features and their structural relationship as determined by molecular networking, Cytoscape (version 3.9.0) was used (42). Random forest classification analysis (43) was used to find the metabolites that were perturbed the most during infection at different stages (1,000 trees). 3D ‘ili models (https://ili.embl.de/) were used to generate 3D models of metabolome perturbation and parasite burden in different organs (44). The 3D model was adapted from the model built in Hossain et al. (28) to add heart, intestinal contents, liver, and quadriceps, using Meshmixer, as described in Dean et al. (27). Undetermined values (below the limit of detection by qPCR) for parasite burden were substituted with the lowest number of parasites detected in each organ divided by five. Median distance correlation plots were generated using ggplot2 (version 3.5.1), with linear regression R^2^ and equations determined in Excel. PERMANOVA, univariate tests (Mann-Whitney U-test), and Spearman correlation *P*-values were corrected using Benjamini-Hochberg false discovery rate (FDR) method in R (version 4.5.0) using the p.adjust() function with the method set to BH. Mann-Whitney tests were reported with common language effect size (CLES). Spearman correlations were reported with 95% confidence intervals.

### Assessment of parasite burden

DNA was isolated from 20 mg of tissue homogenate in water, generated as described above. Genomic DNA was isolated with DNeasy Blood & Tissue Kit (Qiagen# 69506/69581) per the manufacturer’s instructions. Parasite burden was measured by quantitative RT-PCR using 100 ng DNA per reaction on a QuantStudio 6 Flex (Applied Biosystems) using Thermo Fisher (cat # 4444557) TaqMan Fast Advanced Master Mix as previously described (16). The following Taqman primer/probes were used in a multiplex reaction. *T. gondii* 529 bp Repeat Element (RE): *forward:* 5′-CACAGAAGGGACAGAAGTCGAA-3′; *reverse:* 5′-CAGTCCTGATATCTCTCCTCCAAGA-3′; *probe:* 5′-CTACAGACGCGATGCC-3′ (IDT). This was normalized to mouse beta-actin using the validated probe: Mm02619580_g (Thermo Fisher Scientific).

### Tissue cytokine measurement with enzyme-linked immunosorbent assay

Remaining tissue homogenate in water (as described above) was assayed for levels of IFN-γ (Thermo Fisher 88-7314) or IL-1β (Thermo Fisher 88-7013) following the manufacturer’s instructions. Cytokine concentration was determined by standard curves and reported as pg/mL tissue homogenate. Enrichment was determined by unpaired t-test with two-stage set up method of Benjamini, Kreifer, and Yekuteli FDR using Graphpad Prism, comparing uninfected versus infected animals within each tissue, time point, and/or genotype. For tissues where cytokines were not detected (ND) in some or all animals, Fisher’s test was performed.

### 16S rRNA gene amplicon sequencing

The contents from the small intestine, cecum, and large intestine were homogenized in 175 µL of water per 50 mg of sample, aliquoted, and stored at −80°C. DNA was extracted from the intestinal contents using the Qiagen DNeasy PowerSoil Pro Kit (cat #47016) per the manufacturer’s instructions. 16S ribosomal DNA gene sequencing was performed by Novogene. Briefly, the V4 hypervariable region of the 16S rRNA gene was amplified using barcoded primers 515F (GTGCCAGCMGCCGCGGTAA) and 806R (GGACTACHVGGGTWTCTAAT) (45). Amplicons were visualized on a 2% agarose gel, quantified with real-time PCR, and pooled in equimolar concentration followed by end-repairing, A-tailing, and Illumina adapters. Libraries were sequenced on an Illumina NovaSeq 6000 platform to generate 250 bp paired-end raw reads. Two negative controls were included during sample processing and sequencing.

### 16S rRNA gene bioinformatics analysis

The 16S rRNA gene sequence with barcode and primer removed was analyzed with DADA2 Workflow for Big Data (46) and dada2 (v 1.26.0) (47). Forward and reverse reads were trimmed using lengths of 150 bp, filtered to contain no ambiguous bases, and contained a minimum quality score of 2. Reads were assembled and chimeras were removed per dada2 protocol.

Taxonomy was assigned to each amplicon sequence variant (ASV) using a combination of the SILVA v128 database (48) and the RDP naïve Bayesian classifier as implemented in the dada2 R package (47). Read counts for ASVs assigned to the same taxonomy were summed for each sample. Alpha diversity of microbiome samples was evaluated using the Shannon alpha diversity index. Beta diversity of the microbiome was measured using Bray-Curtis dissimilarity measures on the basis of relative abundance data. The statistical significance of alpha diversity was determined by pairwise comparisons using the Wilcoxon rank sum test with continuity correction, while the significance of beta diversity and relative abundance of each family were measured by permutational multivariate analysis of variance (PERMANOVA). Comparisons of microbiota families in uninfected and infected samples were performed with Welch’s unpaired *t*-test.

### mmvec

For each intestinal content and infectious stage, a table of all detected metabolites and of the bacterial families with abundance >5% was subjected to microbe-metabolite vectors analysis (mmvec) (49), producing conditional probabilities between metabolites and microbiome families. In R (RStudio, version 2023.03.0), non-annotated metabolites and metabolites with mean decrease accuracy lower than 1 from random forest analysis were subsequently excluded from the conditionals file. The subsetted conditionals file was then subjected to Principal Component Analysis (PCA) and visualized using the pca3d package (version 0.10.2) in R.

### MicrobeMASST

To identify a putative microbial origin for metabolites perturbed by infection, MicrobeMASST, an open-source tool integrated into the Global Natural Products Social Molecular Networking (GNPS) platform, was used. MicrobeMASST allows the comparison of experimental tandem mass spectrometry (MS/MS) spectra against a comprehensive GNPS database, which contains spectra derived from bacterial, fungal, and archaeal monocultures (50). For this analysis, MS/MS spectra were submitted to the MicrobeMASST interface. The tool performed a spectral similarity search, comparing each input spectrum to the GNPS repository to identify potential microbial sources. The search results provided a list of microbial strains that produced metabolites with similar MS/MS spectral profiles to those detected in our data set. All spectra were processed using default parameters in MicrobeMASST, except for the following: *m/z* tolerance = 0.02, precursor *m/z* tolerance = 0.02, and min matched signals = 4.

## RESULTS

### Localized metabolic impact of *T. gondii* infection is not directly explained by current local tissue parasite burden

Infectious disease processes are highly localized, with differential sensitivity to pathogen colonization, damage, and recovery processes depending on the organ and tissue (51). Prior studies evaluating the consequences of *T. gondii* infection on metabolism have focused on *in vitro* culture, or *in vivo* analysis of serum or a few tissue sampling sites (12, 14, 19, 20, 52, 53). We therefore sought to generate a systematic understanding of spatial metabolic alterations during acute and chronic *T. gondii* infection. Spatial metabolomic analysis (“chemical cartography”) (54) was performed on eleven sampling sites, selected using the following rationale. Cardiac muscle (site 1) and skeletal muscle (site 2) are major sites of cyst burden during chronic infection. In addition, skeletal muscle (site 2) and liver (site 3) are central metabolic tissues that progressively waste during *T. gondii* infection-induced chronic cachexia (18, 55). During acute *T. gondii* infection, parasites and gut commensal bacteria translocate to the liver, driving Toll-like receptor activation and inflammation (56–58). *T. gondii* interacts with the duodenum (site 4), jejunum (site 5), and ileum (site 6) in the first days post-infection, with highest burdens in the distal small intestine (59). Although *T. gondii* does not infect or directly induce tissue damage to the cecum (site 7) or large intestine (site 8), these organs support the largest population and diversity of gut commensal bacteria (60, 61). Systemic inflammation can shift gut microbial populations and their metabolic output (62). In addition, we sampled the contents of the small intestine (site 9), cecum (site 10), and large intestine (site 11) to determine whether metabolites identified in host tissues were unique or related to any of these gut microbial niches. Each site was sampled in acute (15 days post-infection) and chronic (50 days post-infection) infection in WT C57BL/6 mice infected with *T. gondii* and compared to uninfected littermate tissues harvested on the same day (Fig. S1).

**Fig 1:**
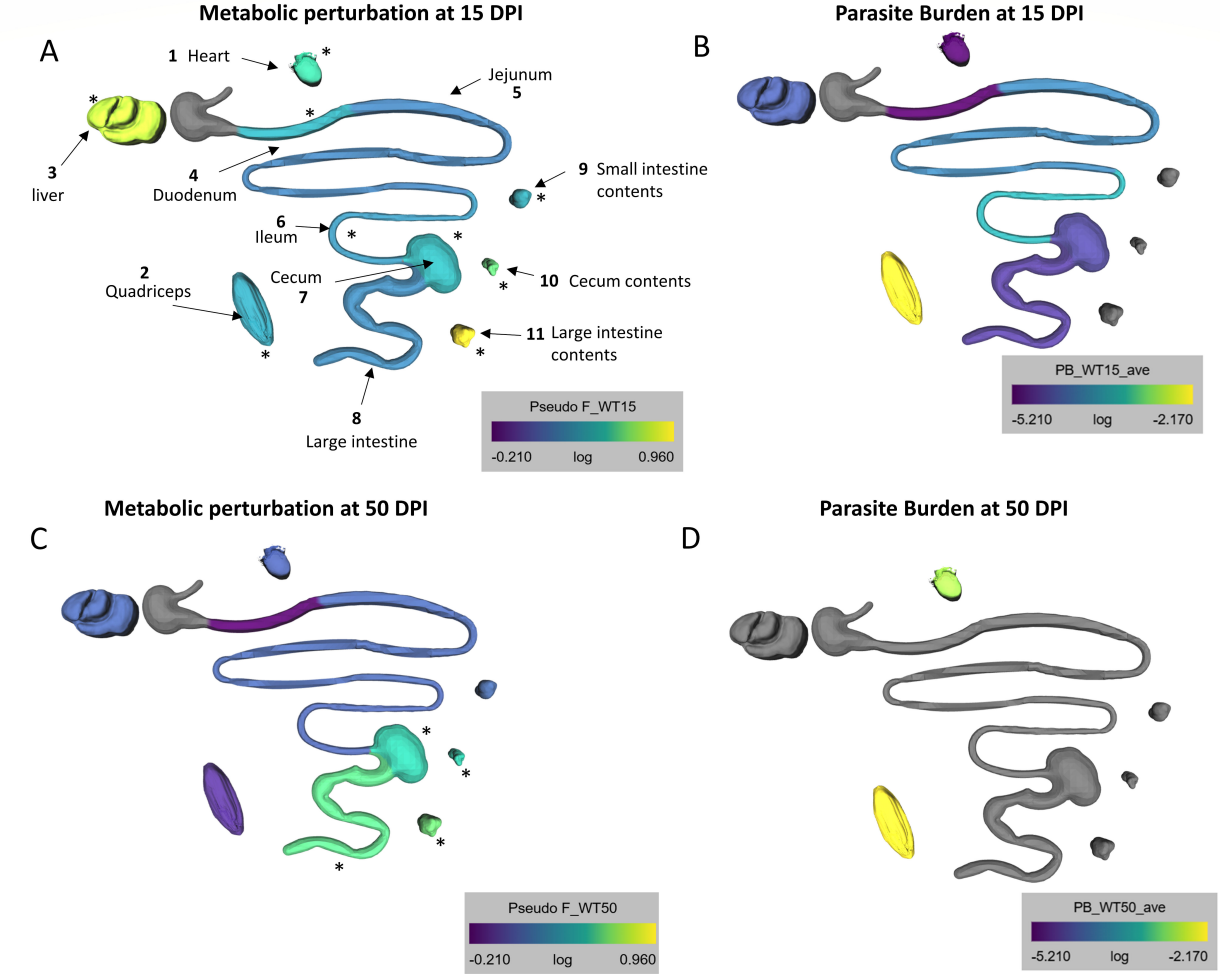
Spatial mismatch between metabolic perturbation and parasite burden at acute and chronic stages of infection. C57BL/6 mice were infected perorally with 20 Me49 *T. gondii* cysts and tissues were dissected at 15 days post-infection (**A and B**) or 50 days post-infection (**C and D**). The metabolic perturbation in each organ is represented as pseudo-F from pairwise PERMANOVA test between infected and uninfected samples at 15 days post-infection (**A**) or 50 days post-infection (**C**). The pseudo-F reflects the effect size when comparing infected vs uninfected groups. Its calculation considers within-group distances (infected vs infected or uninfected vs uninfected) and between-group distances (infected vs uninfected), number of samples, and number of groups. Average parasite burden in each organ was determined at 15 days post-infection (**B**), and average parasite load was surveyed in the cardiac muscle and skeletal muscle at chronic infection (**D**). Gray color denotes organs where metabolites and/or parasite burden were not analyzed. WT 15 day uninfected *N* = 10, 15 day infected *N* = 9, 50 day uninfected *N* = 10, 50 day infected *N* = 7.

In the acute stage of infection in WT mice, the greatest degree of metabolic alteration was in the large intestine contents (FDR-corrected *P* = 0.0044, pseudo-F = 9.165), liver (*P* = 0.001, pseudo-F = 6.148), cecum contents (FDR-corrected *P* = 0.0037, pseudo-F = 4.205), and heart (FDR-corrected *P* = 0.0037, pseudo-F = 3.563), followed by the cecum (FDR-corrected *P* = 0.0044, pseudo-F = 2.650) and duodenum (FDR-corrected *P* = 0.017, pseudo-F = 2.455) (Fig. 1A). This pattern was somewhat unexpected, since some of the tissues with large and significant metabolic perturbation, including the colon and cecum, are not sites of significant *T. gondii* colonization or infection-induced tissue damage (Fig. 1B; Fig. S2) (59, 61, 63) Indeed, at 15 days post-infection, parasite genomic DNA was highest in the skeletal muscle, ileum, and jejunum with low but detectable levels in the duodenum, heart, and large intestine (Fig. 1B). Of note, signal in the large intestine most likely reflects background parasite material shed from the small intestine migrating through the GI tract (56).

The pattern of overall metabolic perturbation was different in the chronic stage in WT mice compared to the acute stage. The highest metabolic perturbation in the chronic stage was observed in the large intestine contents (FDR-corrected *P* = 0.011, pseudo-F = 4.342), large intestine (FDR-corrected *P* = 0.033, pseudo-F = 3.998), and cecum (FDR-corrected *P* = 0.011, pseudo-F = 3.417), followed by cecum contents (FDR-corrected *P* = 0.011, pseudo-F = 3.402) (compare Fig. 1A through C). The small intestine and liver, which clear infection by the chosen chronic infection time point (22, 55, 63), had re-normalized compared to the acute stage (Fig. 1C). As expected, the skeletal muscle and heart, where parasite load remained high, presented with sustained metabolic perturbation (Fig. 1C and D).

**Fig 2:**
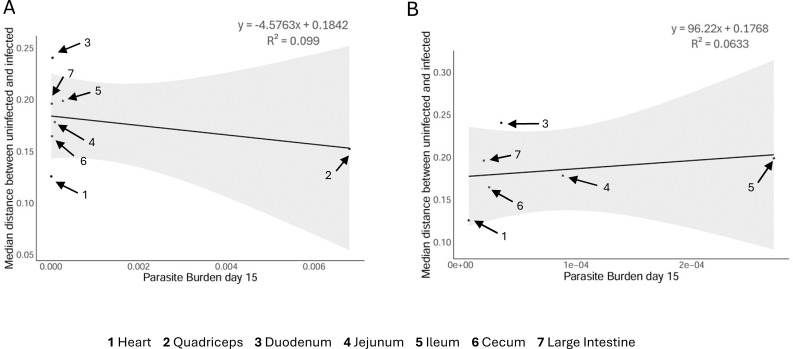
Acute tissue parasite burden and the severity of tissue metabolic perturbation do not correlate. Metabolic perturbation represented as median metabolic distance from pairwise PERMANOVA between infected and uninfected samples (Y-axis) was plotted relative to the average parasite burden in each organ for each tissue in acute infection (**A**) and with the quadricep outlier (much higher parasite burden) removed to confirm that this sample is not skewing the correlation (**B**). The liver data are not displayed to enable comparison of this correlation with the data presented in Fig. 3. The gray shaded region indicates the confidence interval for the linear model.

To further confirm this lack of relationship between local parasite burden and local metabolic change at the time of tissue sampling, we assessed the relationship between median pairwise metabolic distance infected to uninfected samples vs average tissue parasite burden. Strikingly, there was no correlation between the magnitude of metabolite perturbation and the local parasite burden in each organ during the acute stage (Fig. 2).

These data established that the major sites of metabolic perturbation are dynamic from 15 to 50 days post-infection, and that the discordance between local parasite burden at the time of tissue sampling and the degree of metabolic perturbation observed in the acute stage is also apparent at chronic infection timepoints.

To identify metabolites that were differentially regulated in response to infection, random forest analysis was applied (Data set S1). Only a few of the metabolites impacted by infection were correlated with parasite burden, most of which were lipids. Ceramides were negatively correlated with parasite burden in the large intestine. Ceramides regulate cell signaling pathways (64). Ceramide biosynthesis in *T. gondii* has been linked to the parasite’s ability to adapt to different host environments; therefore, this metabolite could be parasite derived (65). Glycerophosphocholines had a mixed profile, with some positively correlated with parasite burden and some negatively correlated in the acute stage. Abnormal phosphatidylcholine ratios could influence energy metabolism and can be a sign of disease progression in metabolic disorders, including atherosclerosis and obesity (66). However, none of these metabolite-parasite burden correlations were significant after correction for multiple hypothesis testing.

### Magnitude of the localized metabolic impact of *T. gondii* infection is partially explained by local tissue inflammation

**Fig 3:**
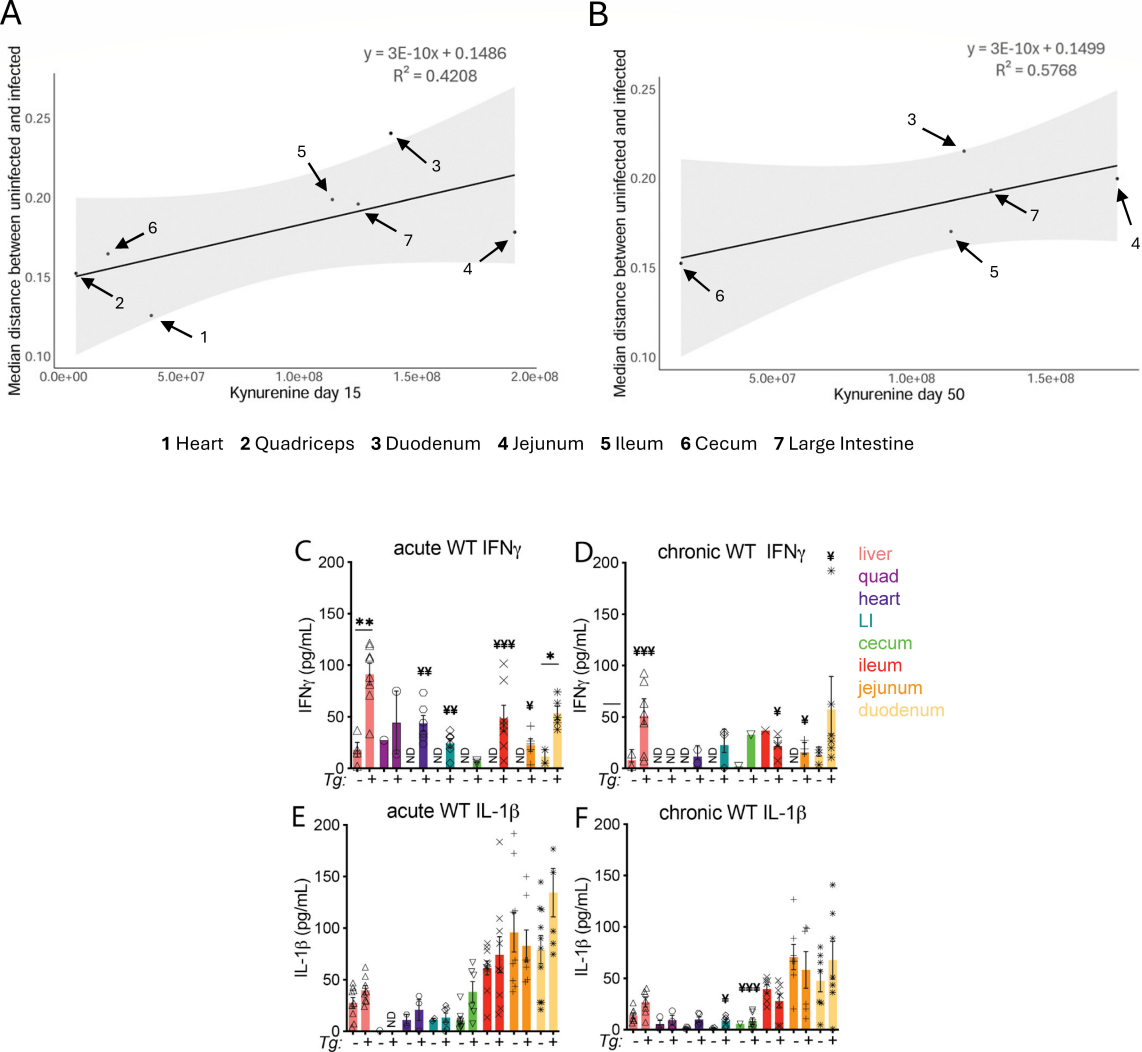
Severity of tissue metabolic perturbation correlates with kynurenine levels. (**A and B**) Median metabolic distance between uninfected and infected tissues relative to median kynurenine levels in acute infection (**A**) and chronic infection (**B**). The gray shaded region indicates the confidence interval for the linear model. Organs where kynurenine was only detected in one sample or fewer are excluded from analysis. Metabolic perturbation is represented as a median metabolic distance from pairwise PERMANOVA. (**C–F**) Analysis of IFN-γ (**C and D**) or IL-1β (**E and F**) levels in tissue homogenate of WT mice. Unpaired T-test using a two-stage setup Benjamini, Krieger, and Yekutieli FDR between uninfected and infected mice for each tissue time point. *P < 0.05, **P < 0.01, and ***P < 0.001. Fisher’s test on the proportion of samples where cytokines were detected comparing infected and uninfected samples within tissues ¥ *P* < 0.05, ¥ ¥ *P* < 0.01, and ¥ ¥ ¥ *P* < 0.001. ND = samples below the detection level of the standard curve. Each symbol represents an individual mouse, where cytokine was detected. WT 15 day uninfected *N* = 10, 15 day infected *N* = 9, 50 day uninfected *N* = 10, 50 day infected *N* = 7.

Having established that shifts in metabolic homeostasis are not strongly explained by concurrent, local *T. gondii* parasite burden, we next assessed whether tissue-specific pro-inflammatory immune responses could be responsible for the observed localized metabolic perturbations. These responses may reflect pathways induced by local parasites, by the killing of parasites previously located at the sampling site, or systemic inflammation. Immune responses can reshape local metabolism, and local immune responses can remain activated even after pathogen clearance (67, 68). Kynurenine is induced by pro-inflammatory signaling and regulates immunity (69). Because we could readily detect it by mass spectrometry in our data set, we used tissue kynurenine levels as a surrogate for the degree of local pro-inflammatory signaling (*m/z* 209.091 RT 0.47 min ([M + H]^+^ adduct) and *m/z* 192.065 RT 0.48 min ([M + H-NH_3_]^+^ adduct)). This annotation was previously validated using pure standards under the same chromatography conditions, at a similar retention time (level 1 annotation confidence according to the metabolomics standards initiative (26, 38); Fig. S7). Kynurenine was significantly increased by infection in seven sampling sites during the acute stage. We observed a moderate correlation between the metabolic impact of infection and kynurenine levels in the acute stage (R^2^ = 0.4208), with a stronger correlation in the chronic stage of infection (R^2^ = 0.5768) (Fig. 3). In contrast to this strong relationship between kynurenine and between-organ differences, kynurenine did not correlate with within-organ differences between mice (Fig. S3).

Metabolites related to inflammation pathways were affected in both acute and chronic stages of infection and correlated with kynurenine levels. For example, lysophosphatidylcholine (LPC) can contribute to inflammation (70). Lyso-PC(16:0) (Spearman correlation coefficient ρ = 0.619, 95% CI [0.374,0.812], FDR-corrected *P* = 0.025) was highly correlated with kynurenine in the ileum in the acute stage of infection. Reduced glutathione (ρ = −0.714, 95% CI [−0.885, −0.425], FDR-corrected *P* = 0.006) and S-hexyl-glutathione (ρ = −0.67, 95% CI [−0.857, −0.332], FDR-corrected *P* = 0.015) were negatively correlated with kynurenine in the heart in acute infection. These metabolites are mostly involved in oxidative stress defense. Beta oxidation-related metabolites, specifically acylcarnitines, were also strongly correlated with kynurenine. For example, acetyl-carnitine was elevated in the heart (ρ = 0.639, 95% CI [0.202−0.818], FDR-corrected *P* = 0.024) while propionylcarnitine (ρ = −0.616, 95% CI [−0.838, −0.243], FDR-corrected *P* = 0.03) and carnitine (ρ = −0.502, 95% CI [−0.735, −0.127], FDR-corrected *P* = 0.09) were negatively correlated with kynurenine in the heart.

Interferon-γ (IFN-γ) is critical for control of *T. gondii* infection and one of several cytokines that can upregulate indoleamine-2,3-deoxygenase, the enzyme that converts tryptophan to kynurenine (71). In acute infection, the majority of infected mice had measurable IFN-γ in the heart and intestinal samples, compared to uninfected mice, where IFN-γ was not detected (Fig. 3C). Moreover, the duodenum and the liver exhibited the highest IFN-γ levels at both acute and chronic time points (Fig. 3C and D). In contrast, kynurenine was not detected in the livers of all but one mouse (Fig. 3A), and the jejunum, which had among the lowest levels of IFN-γ (Fig. 3C), had the highest level of kynurenine (Fig. 3A), indicating that IFN-γ-independent regulation of kynurenine may be occurring during *T. gondii* infection at these and/or other tissue sites. One possible signal is IL-1, which was high in the duodenum and can synergize with IFN-γ to induce kynurenine production (72).

### IL1R signaling contributes to the metabolic impact of *T. gondii* infection

The IL-1 signaling axis has recently emerged as a mechanism regulating disease tolerance in acute *T. gondii* infection. In a lethal model of peroral infection, IL-1R deficiency limited the development of pathological TH17 response in the ileum (73). IL-1R deficiency also increased liver and adipose tissue necrosis in acute infection (16), although these tissues underwent repair and were ultimately protected from fibrotic tissue remodeling (55). These data indicate that protective vs. pathogenic roles for this signaling cascade may depend on the context of the signaling environment and the duration of signaling. In acute and chronic infection, IL-1β levels were greatest in the small intestine, although infection did not significantly impact IL-1β abundance at the time points sampled (Fig. 3E and F). Although overall levels of IL-1β were low in chronic infection in large intestine and cecum samples, the cytokine was detected in significantly more samples in infected mice compared to uninfected animals, consistent with a perturbed inflammatory environment in these tissues (Fig. 3F, Fisher’s test *P* < 0.05). Together, these data are consistent with both the role that the IL-1 axis plays in regulating disease tolerance during *Toxoplasma* infection and its role in regulating interactions with other enteric pathogens (16, 74). To understand how IL-1 signaling impacted metabolic perturbations during acute *T. gondii* infection, we leveraged IL-1R-deficient (IL1RKO) mice.

**Fig 4:**
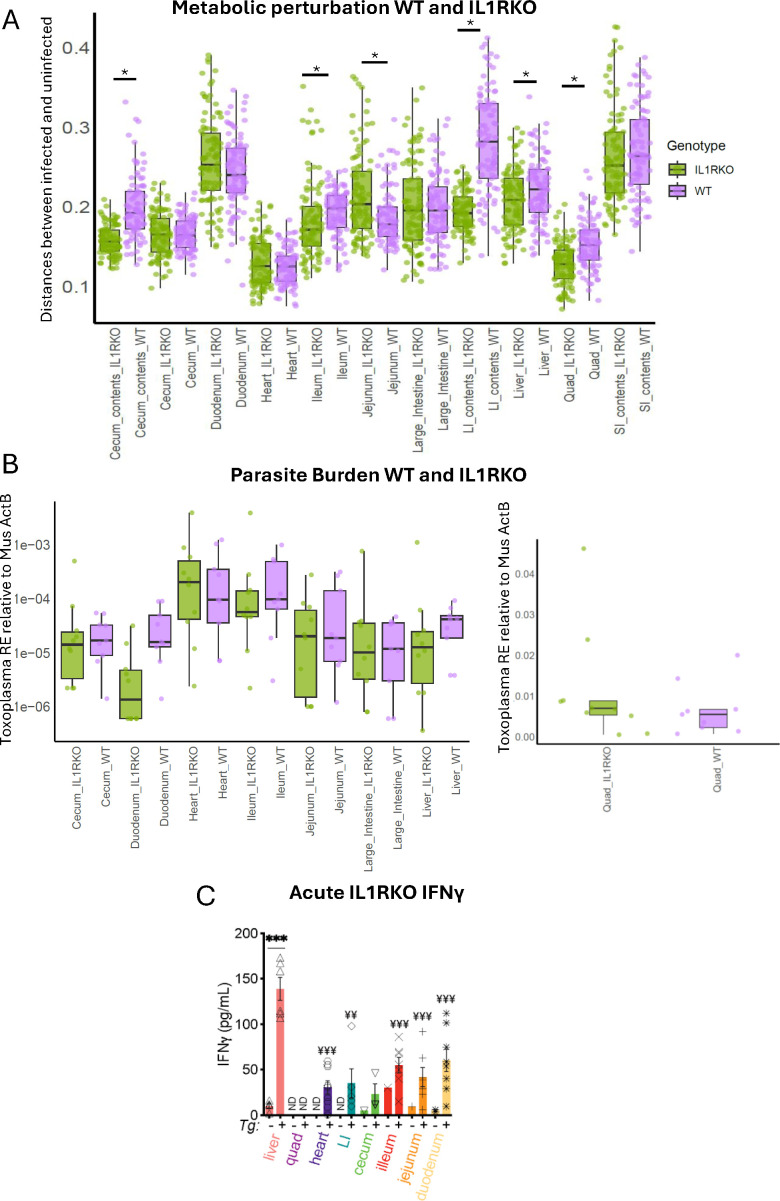
Reduced metabolic impact of *T. gondii* infection in IL1RKO mice. (**A**) Pairwise distances between infected and uninfected samples in the 11 sampling sites in WT and IL1RKO mice. Significant differences using Benjamini-Hochberg FDR-corrected Wilcoxon test between WT and IL1RKO are indicated by asterisks (*). (**B**) Parasite burden at corresponding sites. Quadriceps is plotted separately due to the higher parasite burden, to allow for a clearer visualization of other sites. (**C**) Analysis of Interferon-γ levels in tissue homogenate of IL1RKO mice, as described in Fig. 3. Unpaired T-test using a two-stage setup Benjamini, Krieger, and Yekutieli FDR correction between uninfected and infected mice for each tissue time point. ***P < 0.001. Fisher’s test on proportion of samples where cytokines were detected ¥ *P* < 0.05, ¥ ¥ *P* < 0.01, ¥ ¥ ¥ *P* < 0.001. ND = samples below the detection level of the standard curve. WT 15 day uninfected *N* = 10, 15 day infected *N* = 9; IL1RKO 15 day uninfected *N* = 10, 15 day infected *N* = 10 (**A and B**), *N* = 9 (**C**).

Notably, the metabolome of infected and uninfected samples was more divergent in WT mice than in IL1RKO mice in the large intestine contents (Mann-Whitney FDR-corrected *P* = 1.03E-21, CLES = 0.903), cecum contents (*P* = 6.02E-16, CLES = 0.84), quadriceps (*P* = 4.42E-8, CLES = 0.73), ileum (*P* = 1.16E-3, CLES = 0.637), and liver (*P* = 3.75E-2, CLES = 0.588) with the opposite pattern in the jejunum (*P* = 7.74E-4, CLES = 0.359) (Fig. 4A), even though the parasite burden was statistically similar in IL1RKO compared to WT at these sites (Fig. 4B). Importantly, this pattern was independent of IFN-γ signaling, since IFN-γ levels were significantly enriched in the liver, small intestine, cecum, and large intestinal tissue of IL1RKO-infected mice relative to IL1RKO-uninfected and WT-infected mice relative to WT uninfected (Fig. 3C and 4C) and levels of IFN-γ were statistically similar between genotypes for each tissue.

We then assessed whether the impact of infection on individual metabolites in IL1RKO was higher or lower than observed in WT mice. For the infection-perturbed metabolites, as determined in WT mice by random forest analysis (Data set S1), fold changes were mainly higher in the WT mice in all sampling sites except the cecum contents and large intestine where fold changes were mainly higher in IL1RKO mice. In the heart, there were comparable numbers of metabolites that had higher fold changes in WT and in IL1RKO. In the large intestine contents, for example, the fold change in metabolite peak area between infected and uninfected samples was higher in WT in 57.1% of the 238 infection-perturbed metabolites, compared to 24.8% higher in IL1RKO. In the liver, the fold changes were higher in WT in 45.3% of 254 metabolites and 24.8% in IL1RKO. Additionally, in the ileum, the fold change was higher in WT in 43.2% of 273 metabolites and 20.2% in IL1RKO. The remaining metabolites had opposite directions of change in WT and IL1RKO, and thus fold changes could not be compared. Overall, these results are consistent with a model in which signaling through the IL-1R contributes to the severity of metabolic perturbation in sites that interface directly with the parasite, the small intestine, liver, skeletal, and cardiac muscle, as well as the cecum and large intestine, which are not substantially infected directly.

### Role of the gut microbiome in local tissue metabolic responses to infection

In both WT and IL1RKO mice, significant metabolic impacts of infection were observed in the cecum contents (WT FDR-corrected *P* = 0.0044 Pseudo-F = 4.21 and IL1RKO FDR-corrected *P* = 0.066 Pseudo-F = 1.51) and in intestinal contents (WT FDR-corrected *P* = 0.0044 Pseudo-F = 9.16 and IL1RKO FDR-corrected *P* = 0.0037 Pseudo-F = 2.29). However, kynurenine, IL-1β, and IFN-γ levels were low in the cecum, indicating other mechanisms beyond direct effects of inflammation may be at play. The microbiota is a major metabolic driver, with particularly strong effects on intestinal tissues (75). For example, indirect effects of *T. gondii* infection on the microbiota could be responsible for the metabolic changes observed in the cecum and large intestine. Multiple metabolites identified as impacted by *T. gondii* in this study are or can be of microbiota origin, including amino acid-conjugated bile acids (75) and pantothenate (76) (Data set S1). The function of amino acid-conjugated bile acids is still under investigation; however, some of them are inducers of IL-17 by CD4^+^ T cells. Bile acids can also reshape the gut microbiota composition (77) and have a major impact on metabolism (78, 79). Given the tight connection between inflammation, microbiota, and host metabolism (75, 80), we therefore assessed whether we observed disruptions in the gut microbiota of these mice, and whether these compositional changes could be associated with the metabolic changes we observed, using 16S rRNA gene sequencing, microbe-metabolite vectors (mmvec) analysis (49) and microbeMASST analysis (microbial Mass Search Tool analysis) (50).

**Fig 5:**
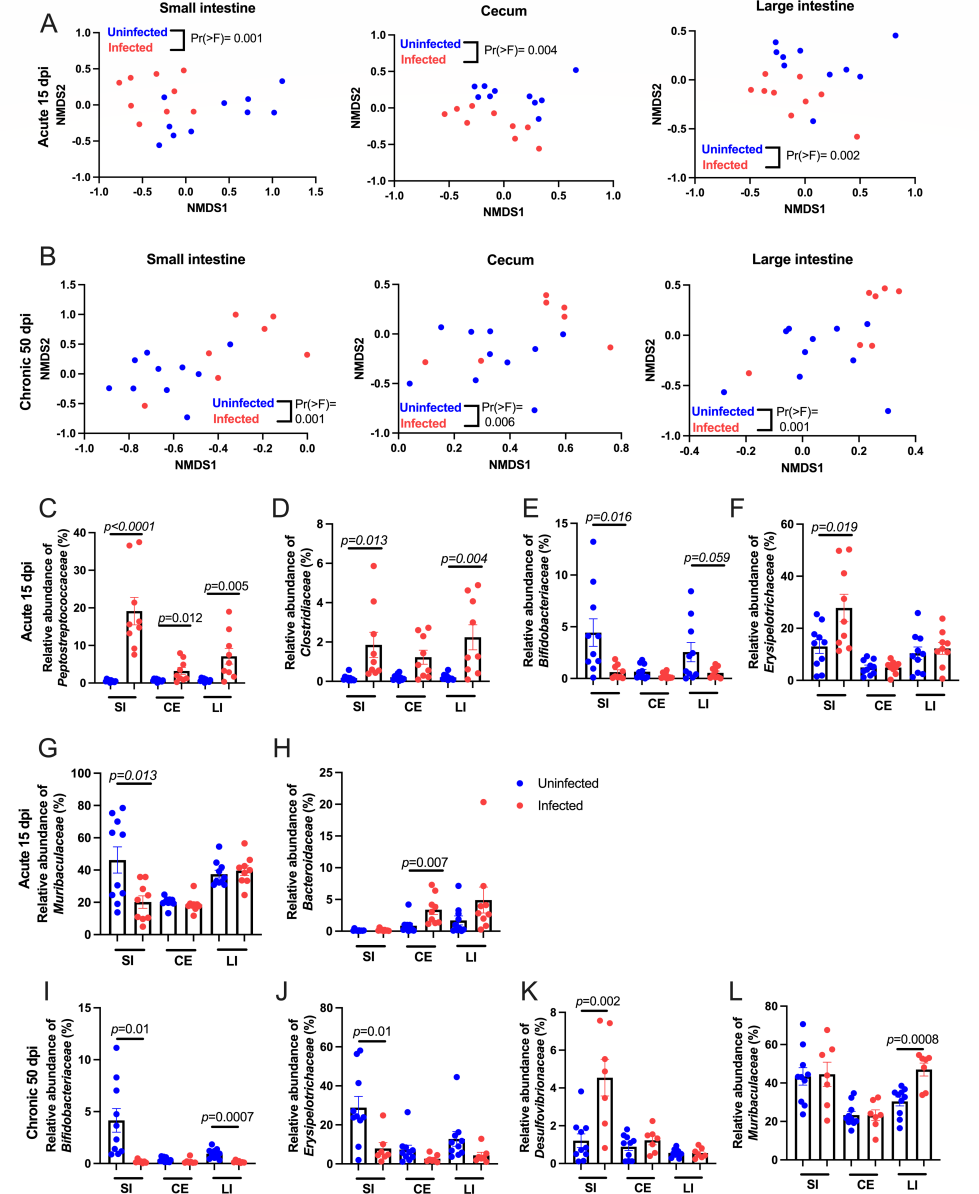
Impact of acute and chronic *T. gondii* infection on the microbiome. (**A and B**) Beta diversity of bacterial components was compared using the Bray’s NMDS analysis of 16S rRNA gene sequencing at 15 days post-infection (**A**) or 50 days post-infection (**B**). Statistical significance was determined by Permutational multivariate analysis of variance (PERMANOVA). (**A**) Small intestine (Pr(>F) = 0.001, R^2^ = 0.5984), cecum (Pr(>F) = 0.004, R^2^ = 0.5727), and large intestine contents (Pr(>F) = 0.002, R^2^ = 0.5616). (**B**) Small intestine (Pr(>F) = 0.001, R^2^ = 0.4824), cecum (Pr(>F) = 0.006, R^2^ = 0.5009), and large intestine contents (Pr(>F) = 0.001, R^2^ = 0.5246). (**C–H**) At 15 days post-infection, the relative abundance of (**C**) *Peptostreptococcaceae* (median difference = 5.283, 95%CI = 1.387 to 13.28), (**D**) *Clostridiaceae* (median difference = 1.136, 95%CI = 0.324 to 4.005), (**E**) *Bifidobacteriaceae* (median difference = −3.015, 95%CI = −6.769 to 0.7419), (**F**) *Erysipelotrichaceae* (median difference = 12.76, 95%CI = −0.5948 to 29.81), (**G**) *Muribaculaceae* (median difference = −25.94, 95%CI = −52.160 to −2.312), and (**H**) *Bacteroidaceae* (median difference = 2.069, 95%CI = 0.069 to 3.994), in the small intestine (SI), cecum (CE), and large intestine (LI) contents from uninfected and infected mice. (**I–L**) At 50 days post-infection, the relative abundance of (**I**) *Bifidobacteriaceae* (median difference = 2.069, 95%CI = 0.069 to 3.994)*,* (**J**) *Erysipelotrichaceae* (median difference = −0.546, 95%CI = −0.116 to −0.012), (**K**) *Desulfovibrionaceae* (median difference = 0.287, 95%CI = −0.4785 to 1.156), and (**L**) *Muribaculaceae* (median difference = 0.089, 95%CI = 0.038 to 0.168) in the intestinal contents from uninfected and infected mice. Error bars represent Welch’s unpaired *t*-test ± SEM and Hodges-Lehmann median differences. 15 day uninfected *N* = 10, 15 day infected *N* = 9, 50 day uninfected *N* = 10, 50 day infected *N* = 7.

Bray non-metric multidimensional scaling (NMDS) analysis showed that the microbial 16S rRNA gene composition in the small intestine, cecum, and colon contents was distinct in uninfected and infected littermates in acute infection (Fig. 5A). The disruption in the microbial composition between infected and uninfected mice remained significant in chronic infection (Fig. 5B). In acute infection, increased *Peptostreptococcaceae* (Fig. 5C) and *Clostridiaceae* (Fig. 5D), and a decrease in *Bifidobacteriaceae* (Fig. 5E) were observed in the small intestine and large intestine contents in acute infection. In addition, there were some infection-dependent changes that were unique to each intestinal tract site. For example, there was a significant increase in small intestine *Erysipelotrichaceae* and a decrease in *Muribaculaceae* (Fig. 5F and G). The cecum had an increase in *Bacteroidaceae* (Fig. 5H). In chronic infection, there were still microbial species that were significantly differentially enriched in the small intestine contents, including *Bifidobacteriaceae* (also in the colon contents) (Fig. 5I), *Erysipelotrichaceae* (Fig. 5J), and *Desulfovibrionaceae* (Fig. 5K).

**Fig 6:**
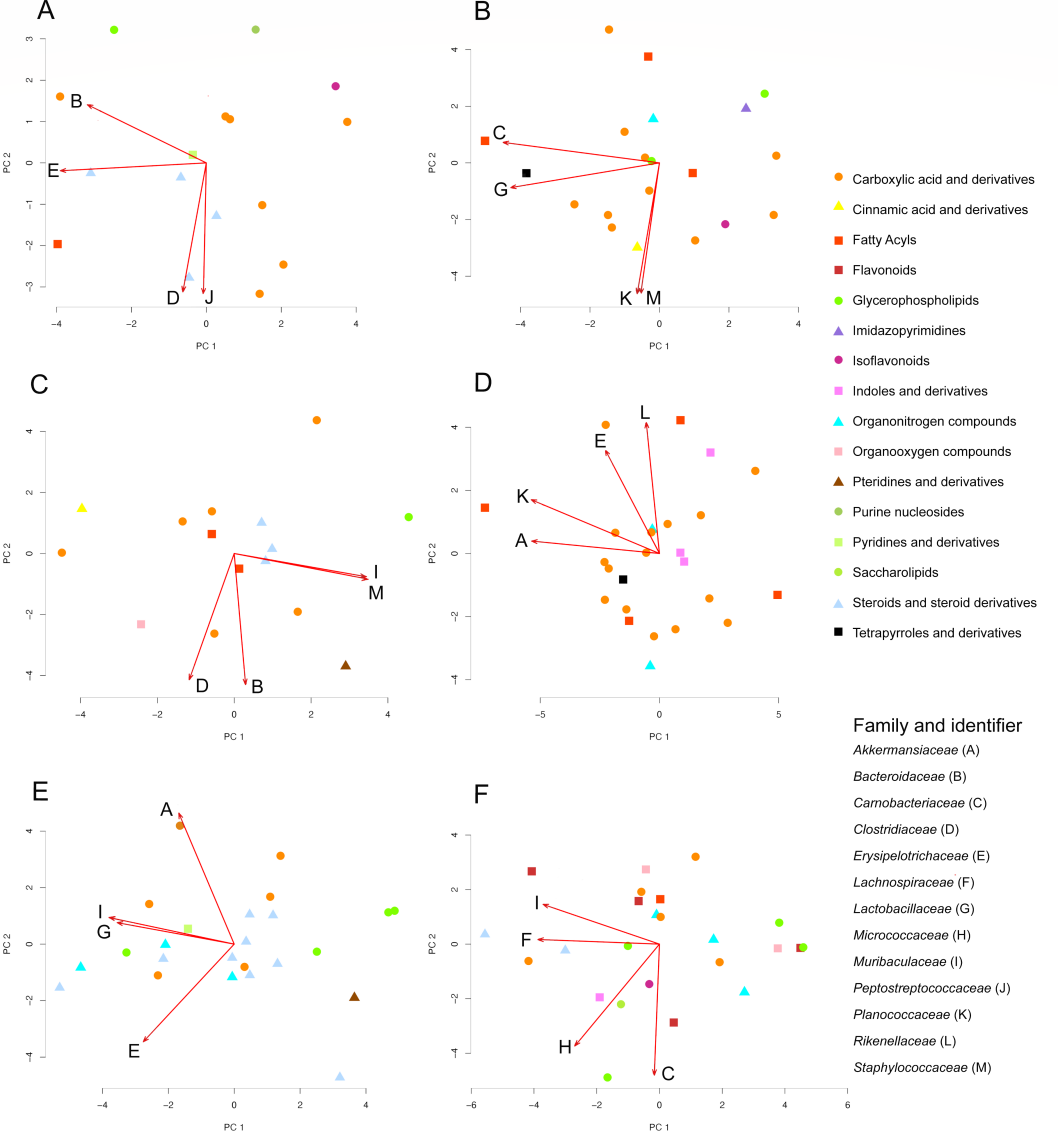
Co-occurrence between the metabolites most perturbed by infection and gut microbiome families in GI tract contents. mmvec analysis was applied to the most abundant microbial families (>5% abundance) and on the metabolite peak areas, followed by PCA analysis. Only metabolites with a random forest mean decrease accuracy > 1 and with annotations are displayed. (**A**) Cecum contents in the acute stage. (**B**) Cecum contents in the chronic stage. (**C**) Large intestine contents in the acute stage. (**D**) Large intestine contents in the chronic stage. (**E**) Small intestine contents in the acute stage. (**F**) Small intestine contents in the chronic stage. Red arrows, four most influential microbiome families. Capital letters, identifiers for each microbiome family. 15 day (acute) uninfected *N* = 10, 15 day infected *N* = 9, 50 days (chronic) uninfected *N* = 10, 50 day infected *N* = 7.

To predict candidate bacterial origins for infection-impacted metabolites, we evaluated co-occurrence patterns between the 16S rRNA gene sequencing data and metabolite data using microbe-metabolite vectors (mmvec) analysis (49). Multiple patterns of co-occurrence were observed, such as between steroids and *Clostridiaceae* family members, *Erysipelotrichaceae* family members, and *Peptostreptococcaceae* family members in cecum contents during acute infection or with *Muribaculaceae* and *Staphylococcaceae* (lower in infected versus uninfected, FDR-corrected *P* = 0.02) family members in the large intestine contents during acute infection. In the small intestine contents, *Lachnospiraceae* and *Muribaculaceae* showed opposite occurrence patterns to organonitrogen compounds during chronic infection (Fig. 6).

To identify infection-impacted features with a candidate microbial origin, we performed microbeMASST analysis. MicrobeMASST is an open-source tool within the GNPS environment that matches experimental tandem MS spectra to spectra within the GNPS repository, which were retrieved from bacterial, fungal, and archaeal monocultures (50). Notably, the cecum contents in the chronic stage had the highest percentage of microbeMASST matches (62.8% matches). The main chemical families with matches were phospholipids, amino acids, and peptides. The lowest percentage of microbeMASST matches was observed in the liver in the acute stage of infection (45.7% matches, Data set S1). Corresponding findings were observed for several features in the cecum, large intestine, and small intestine contents between mmvec and microbeMASST analysis. For example, in the cecum contents at day 15, *m/z* 785.589 retention time (RT) 2.78 min had a co-occurrence with *Peptostreptococcaceae,* which was supported with a microbeMASST match to *Peptostreptococcus* genus. In the large intestine contents at day 50, for metabolite feature *m/z* 187.144 RT 0.45 min, mmvec found an *Akkermansiaceae* co-occurrence and microbeMASST found a match to *Akkermansia muciniphila*. In the small intestine contents at day 15, metabolite feature *m/z* 175.118 RT 0.26 min had an mmvec match to the *Lactobacillaceae* family, and microbeMASST had several matches to genera within this family. At day 50, for metabolite feature *m/z* 205.096 RT 3.47 minutes, mmvec found co-occurrence with *Micrococcaceae*, supported by a microbeMASST matches to *Micrococcus luteus*. The consistent results from both microbeMASST and mmvec analyses support a microbial origin for these infection-impacted metabolites.

## DISCUSSION

Metabolic changes in association with infectious diseases have been extensively studied (81–83) and demonstrated to play a causal role in disease progression (28, 84, 85). Pathogen-derived metabolites, damage-induced signals, microbiome-derived molecules, and immune-mediated metabolic responses have all been implicated as regulators of these adaptive and maladaptive metabolic changes (11). However, their relative importance across organs in driving overall metabolic changes is not well characterized. Here, using *T. gondii* infection as a model, we assessed the association of tissue site, local tissue parasite burden at the time of sample collection, local tissue immune responses, infection duration, and microbiome composition with tissue metabolic responses. *Toxoplasma* has a well-studied dissemination pattern. In the first weeks of infection, the parasites invade and replicate in foci along the jejunum and ileum and disseminate to the mesenteric lymph nodes, spleen, and liver (22, 57, 59). Parasites are detected in adipose tissue, liver, and lung, peaking between 9 and 14 days (22, 86, 87). Parasites are eventually cleared from these tissues by 4 weeks post-infection, at which point the highest burden is observed in the central nervous system, cardiac, and skeletal muscle tissues. In contrast, the cecum and colon remain unaffected (61). We observed a limited contribution of local tissue parasite burden to metabolic changes as measured at both the acute and chronic times of sample collection (Fig. 1). This observation concurs with our findings with the parasites *Trypanosoma cruzi* (28, 67, 88) and *Leishmania major* (89), and in SARS-CoV-2 infection (90). In contrast, in a mouse model of acute influenza A virus infection, a positive correlation was observed between metabolic perturbation and tissue viral load (26). As in these other systems, however, our findings do not preclude that prior pathogen colonization initiated these observed metabolic changes, even if they are maintained in the absence of parasite persistence. For example, although parasite load in the liver and adipose tissues was undetectable by PCR at 5 weeks post-infection (Fig. 1), these tissues presented with significant perivascular fibrosis at this time (55). Several groups have detected microbial translocation from the gut to the lymph nodes and liver following oral infection with the parasite, which could be alternative sources of inflammation (Fig. 3D) and metabolic dysbiosis in tissues that interface with blood or lymph downstream of the gut (56–58).

Immune factors may indeed be responsible for maintaining metabolic impact at sites of prior parasite burden. Kynurenine, a proxy for tissue inflammation, was positively correlated with metabolic impacts of infection, unlike parasite load (Fig. 3), suggesting a dominant regulatory role of non-resolving inflammation over direct parasite effects. IFN-γ is well established to positively regulate indoleamine 2,3-dioxygenase during *T. gondii* infection, starving the parasite of tryptophan while generating kynurenine as an enzymatic output (91, 92). Interestingly, the liver, which had high IFN-γ levels, had levels of kynurenine that were undetectable in the majority of mice, while some of the gastrointestinal tissues with the lowest IFN-γ (cecum and jejunum) had the most robust increase in kynurenine. These data, and the observation that kynurenine levels across organs did not correlate with parasite DNA levels, suggest that other signals, possibly including IL-1 signaling, and potential shifts in microbial PAMPs may be more potent regulators of kynurenine in this context (93–95). Immunity and metabolism are tightly interlinked (96). Many of the infection-impacted metabolites correlated with kynurenine are known to intersect with immunity, including lysophosphatidylcholines in the ileum during acute infection, hemin in the large intestine, ileum, and jejunum in the acute stage of infection, and glutathione in the heart in acute infection. LPC can increase chemokines, which attract inflammatory cells and increase the release of inflammatory mediators, including IFN-γ (70). Hemin is an activator of the antiviral, antioxidant, and anti-inflammatory heme oxygenase-1 (HO-1) enzyme (97). HO-1 provides cytoprotection against inflammatory and oxidative injury (98). In COVID-19 viral infection, the HO-1 enzyme exerts antiviral properties by interfering with the IFN pathway (98). The glutathione system is a robust antioxidant system in the heart responsible for scavenging ROS (99, 100). Decreased levels indicate antioxidant usage corresponding to increased inflammation and could indicate cardiovascular damage (101). This is consistent with observations that ROS production is significant in *T. gondii* infection (101, 102). Sampling earlier time points, where inflammatory signals are more ubiquitous across infected samples, will be useful for identifying correlations between cytokine levels and individual metabolites.

We also used IL1R KO mice to demonstrate that IL1 signaling is responsible for some of the metabolic effects of infection (Fig. 1). Although IL-1β levels in total tissue lysate were low, this was not surprising at 2 and 5 weeks post-infection. Alarmins are potent, local mediators of inflammation and tissue repair, and sustained, systemic release of alarmins is a signature of fatal inflammation (103). Neutral sphingomyelinase expression is regulated by IL-1R accessory protein signaling (104), which promotes diacyl glyceride and ceramide biosynthesis directly. In this study and our previous report (19), we observed significant changes in sphingolipid metabolism during infection. Our data are consistent with a model where the IL-1 axis may impact the inflammatory metabolic state of host tissues more widely and/or for a longer duration than direct signal transduction response. A similar framework will be pursued in future experiments, leveraging KO mice and cytokine blockade, to elucidate the contribution of other immune signals in the metabolic impacts of *T. gondii* infection.

The large intestine contents, large intestine, and cecum were the most metabolically perturbed tissue sites during chronic infection (Fig. 1C). Metabolic perturbation can be regulated by commensal microbes directly through pathogen-associated molecular pattern signaling and microbial metabolites, or indirectly through changes in nutrient availability and cytokine signaling (105). Although overall titers were low in the large intestine and cecum, IL-1β was detected in significantly more infected animals than uninfected (Fig. 3F). IL-1β transcript expression is NFkB-dependent, driven by toll-like receptor signaling, suggesting that the observed shifts in commensals at chronic infection may regulate sustained inflammation in these tissues (74). Microbiota play an important role in regulating the intestinal permeability and inflammatory tone of the gut and other tissues. Peroral infection with *T. gondii* leads to a reduction in commensal diversity during acute infection and outgrowth of Gram-negative species associated with lethal ileitis (56, 106). Non-lethal infection has shown that dysbiosis persists into chronic infection, after inflammatory cytokines and tissue pathology in the small intestine have returned to baseline levels (22, 107). Similar to previous reports, we found that several microbial families associated with pathology were enriched in acute and/or chronic infection, including *Clostridiaceae* and *Enterobacteriaceae* (22, 56, 108). However, previous studies have exclusively evaluated fecal microbes. Our assessment of the small intestine and cecum contents revealed a new role for the anaerobic, Gram-positive family *Peptostreptococcaceae,* which were more highly enriched in the small intestine contents than other sites (Fig. 5C). *Peptostreptococcaceae* and *Clostridiaceae* (Fig. 5C and D) have been associated with frailty and muscle wasting in aged human participants in the BIOSPHERE study (109). Unlike the BIOSPHERE study, we found a reduction in the abundance of *Bifidobacteriaceae* in the small and large intestine contents in acute infection (Fig. 5E). This anaerobic Gram-positive population is involved in oligosaccharide metabolism and has been explored as a probiotic treatment for ulcerative colitis and inflammatory bowel syndrome (110, 111). As expected, the microbial populations were divergent in controls associated with 15 days post-infection and 50 days post-infection populations, which may be related to cohort and/or age-related differences in each cohort (Fig. S4). Despite this natural variation, *Erysipelotrichaceae* was increased in the small intestine during acute infection (Fig. 5F) but significantly depressed during chronic infection (Fig. 5J) compared to uninfected controls. This family includes highly immunogenic species which are positively correlated with dietary fat intake and obesity (112–114).

A possible microbiome source for multiple metabolites impacted by infection was identified in this study, although many of these metabolites do not yet have a known function. The carboxylic acids chemical family, in particular, had strong co-occurrence with influential microbe families in all three gastrointestinal organ contents at both acute and chronic stages. The availability of amino acids affects gut microbiome growth, and the gut microbiome affects amino acid absorption and metabolism (115–117). Amino acid auxotrophies of *T. gondii* can be another reason for amino acid perturbation during toxoplasmosis (118). Future work will build on these results using microbiota depletion (e.g., using antibiotics treatment) and reconstitution studies.

In summary, our study provides valuable insights into the metabolic perturbations induced by *T. gondii* infection across organs and potential mechanisms underlying the observed changes. Future research should focus on elucidating the functional consequences of these perturbations, further exploring their relationship with immune responses, and investigating the effect of metabolic alteration on the gut microbiome and downstream changes in host metabolism during *T. gondii* infection.

## Data Availability

Metabolomics data were uploaded to MassIVE (massive.ucsd.edu), accession number MSV000095605. Molecular networking jobs are accessible through these links: https://gnps.ucsd.edu/ProteoSAFe/status.jsp?task=eaa0f5e4ac6649099029626861b8d843 (feature-based molecular network) and https://gnps.ucsd.edu/ProteoSAFe/status.jsp?task=9391d20cc6a64315994fc21142fd28a1 (MolNetEnhancer). Representative code used to generate manuscript figures has been deposited at https://github.com/CEmiddleton/McCall-lab/tree/main/Toxoplasma. 16S ribosomal RNA gene sequencing metagenomic data have been submitted to GenBank (NCBI) under accession number PRJNA1198079 and will become publicly available once accession formalization resumes.

## SUPPLEMENTAL MATERIAL

Supplemental data set msystems.01126-25-s0001.xlsb Data set S1.

Supplemental material msystems.01126-25-s0002.pdf Table S1; Figures S1 to S7.

ASM does not own the copyrights to Supplemental Material that may be linked to, or accessed through, an article. The authors have granted ASM a non-exclusive, world-wide license to publish the Supplemental Material files. Please contact the corresponding author directly for reuse.
