# Supplementary material for: Spatially divergent metabolic impact of experimental toxoplasmosis: immunological and microbial correlates
Source: mSystems. 2025 Nov 6;10(12):e01126-25. doi: 10.1128/msystems.01126-25 (PMC12710369; doi:10.1128/msystems.01126-25)
Supplement: Supplemental material — Table S1; Figures S1 to S7. [file msystems.01126-25-s0002.pdf]

Supporting information:

Table S1. MZmine parameters (MZmine2 version 2.53).

Mass Detection	MS1 Noise Level	4.0E5
	MS2 Noise Level	1.00E+03
	Mass Detector	Centroid
ADAP Chromatogram Builder	Min group size in # of scans	5
	Group intensity threshold	4.0E5
	Min highest intensity	1.2E6
	m/z tolerance	0.001 *m/z* or 10 ppm
Chromatogram Deconvolution: local minima algorithm	Chromatographic threshold	20
	Search minimum in RT range (min)	0.2
	Minimum relative height	26
	Minimum absolute height	1.6E6
	Min ratio of peak top/edge	1.0
	Peak duration range (min)	0.01-1.00
	m/z Range for MS2 Scan Pairing (Da)	0.01
	RT Range for MS2 Scan Pairing (min)	0.1

Isotopic Peak Grouper	Retention Time Tolerance (min)	0.1
	m/z tolerance (ppm)	10
	Monotonic Shape	Yes
	Maximum Charge	3
	Representative isotope	Lowest *m/z*
Join aligner	*m/z* tolerance (ppm)	10
	m/z to RT weight	10000 to 10
	Retention Time Tolerance (min)	0.32
Row filtering	Retention Time	0.20-7 min
	Remove previous peak list	Disabled
	Keep only peaks with MS2 scan	Enabled
	Minimum peaks in a row	5

Dataset S1. Infection-impacted metabolites and their correlation to parasite burden and kynurenine.

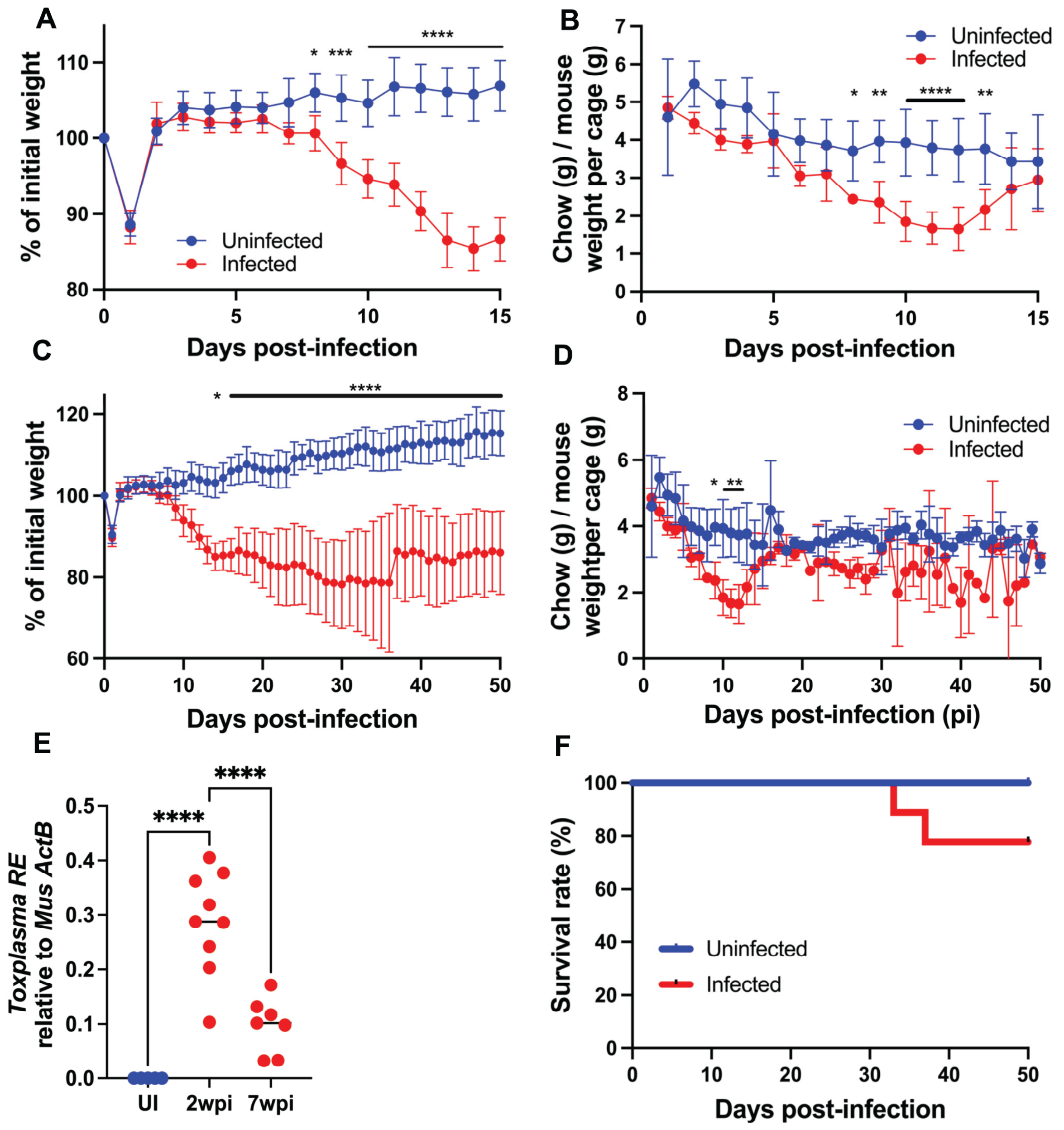

Fig. S1. Impact of infection on mouse weight and food intake in WT mice. C57BL/6J mice were perorally infected with 25 Me49gLuc *T. gondii* cysts of the Me49 strain or mock injected with PBS. (A, C) Change in body weight normalized one day before fasting and peroral infection. (B, D) 24 hour food intake per cage normalized to the pooled weight of all mice in the cage. Unpaired Student's t-test with Sidak method to correct for multiple comparisons. Error bars represent standard error mean. (E) *T. gondii* RE levels relative to host beta-actin in brain

genomic DNA at 15 days or 50 days post-infection. N=10 uninfected mice and N=7-9 infected mice per group pooled from two independent experiments. Statistical significance was determined by One-way ANOVA. (F) Survival curves of mice represented in (C).

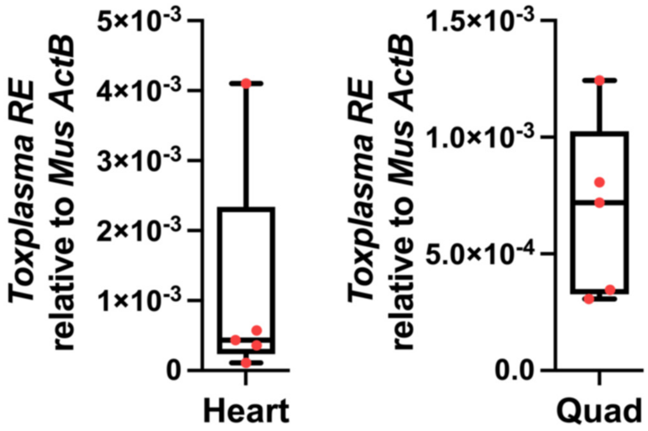

Fig. S2. Parasite burden at 50 days post infection in WT mice as described in Fig. 1. Box and whisker plots represent median, first and third quartile. N=7 infected mice.

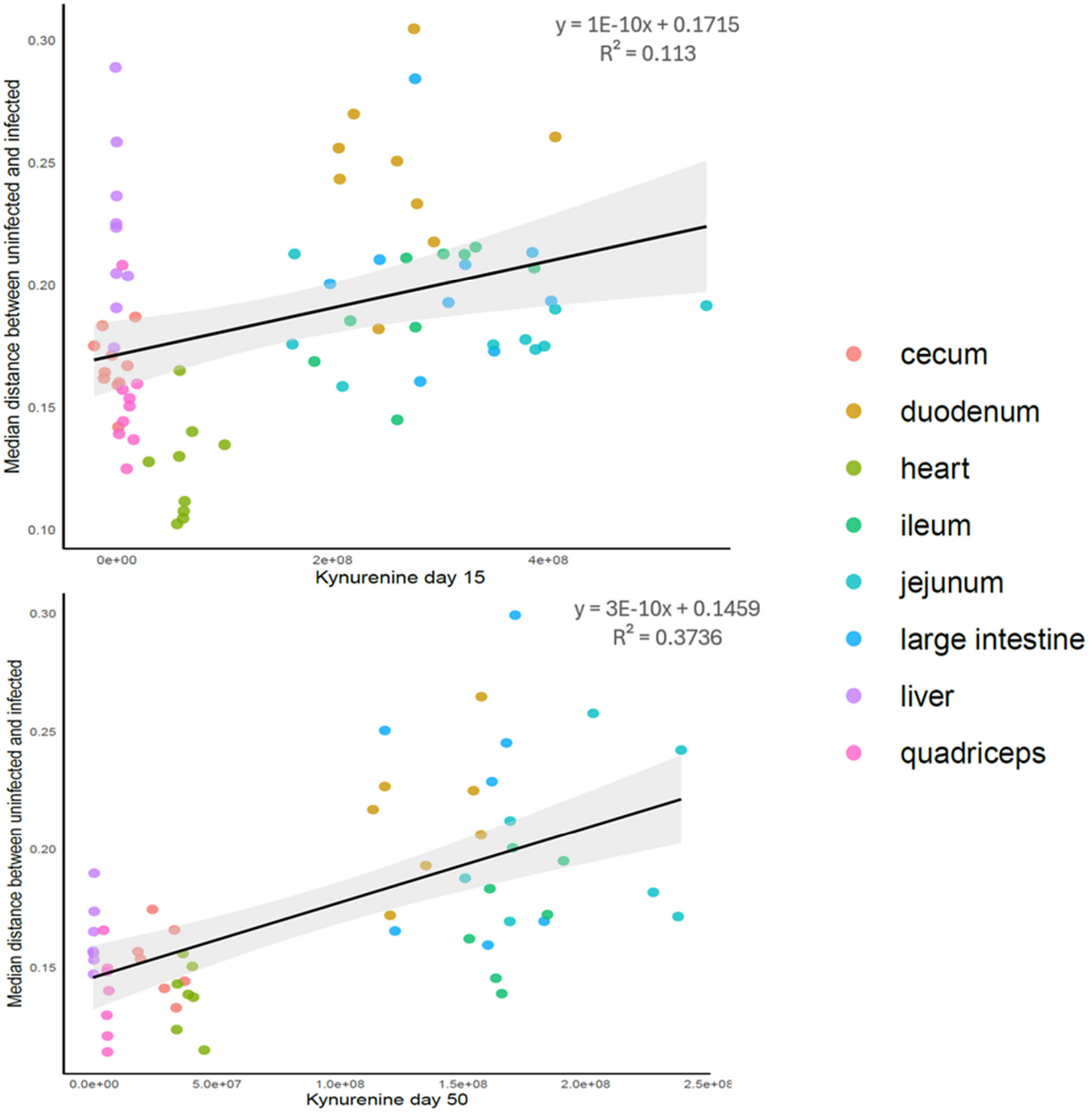

Fig. S3. Lack of correlation between kynurenine levels and metabolic impact on a per-mouse basis. Data represent the correlation between Kynurenine levels measured in each infected mouse, colored by tissue type. 15 days post infection N=9 infected mice, 50 days post infection N=7 infected mice. N=10 uninfected mice.

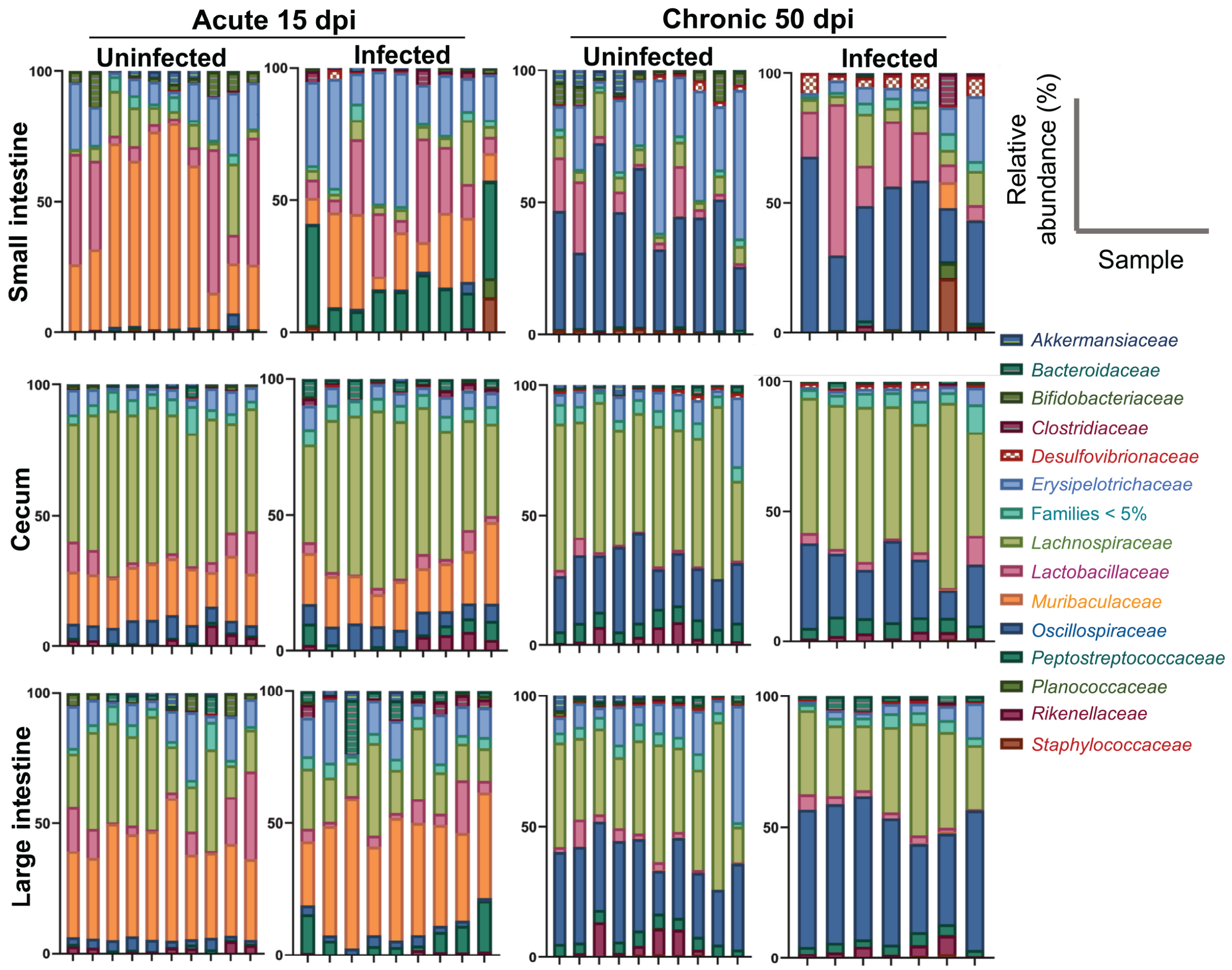

Fig. S4. The relative abundance of small intestine, cecum and large intestine microbial bacteria from uninfected or *T. gondii*-infected mice. C57BL/6J mice were orally infected with *T. gondii* or left uninfected. Bacterial composition at the family level in the small intestine, cecum, and large intestine contents were quantified using 16S sequencing in acute (A) and chronic (B) infection. Each column is an independent mouse.

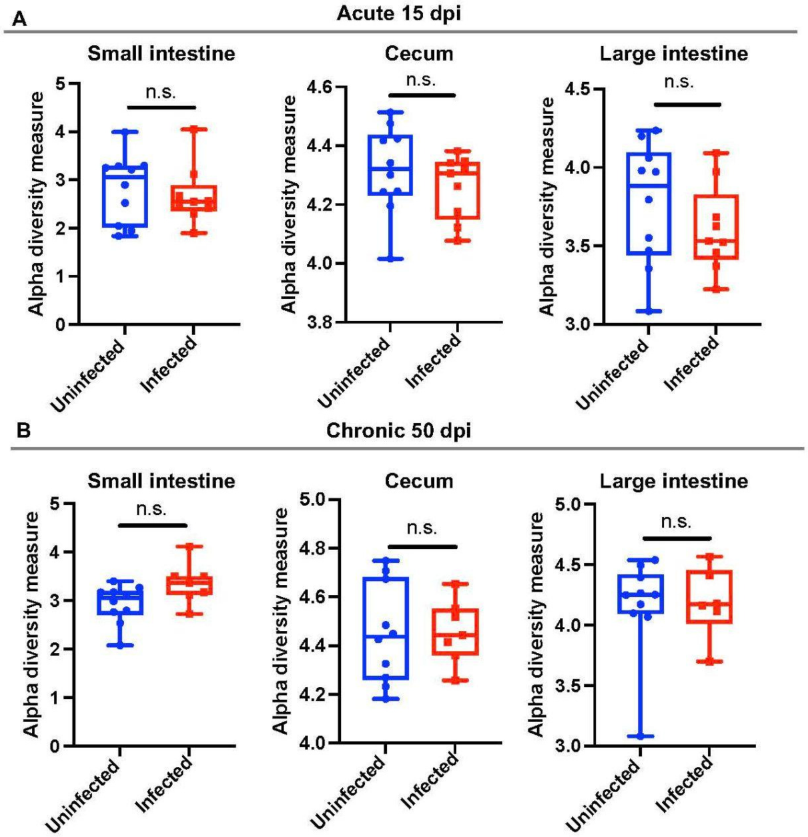

Fig. S5. *T. gondii* infection does not significantly impact intestinal bacterial alpha-diversity at 15 and 50 days post infection. 15 or 50 days post-infection, the contents of small intestine, cecum, and large intestine were collected from *T. gondii*-infected or uninfected WT C57BL/6J mice. The bacterial components in the intestinal contents were analyzed by 16S sequencing. Bacterial alpha diversity in the small intestine, cecum, and large intestine contents was determined using the Shannon index at 15 days post infection (A) or 50 days post infection (B). Max, min and median are presented in each boxplot. Statistical significance was determined by unpaired Student's t-test. Each dot represents an individual mouse. N=10 uninfected, N=9 15 dpi infected, N=7 50 dpi infected.

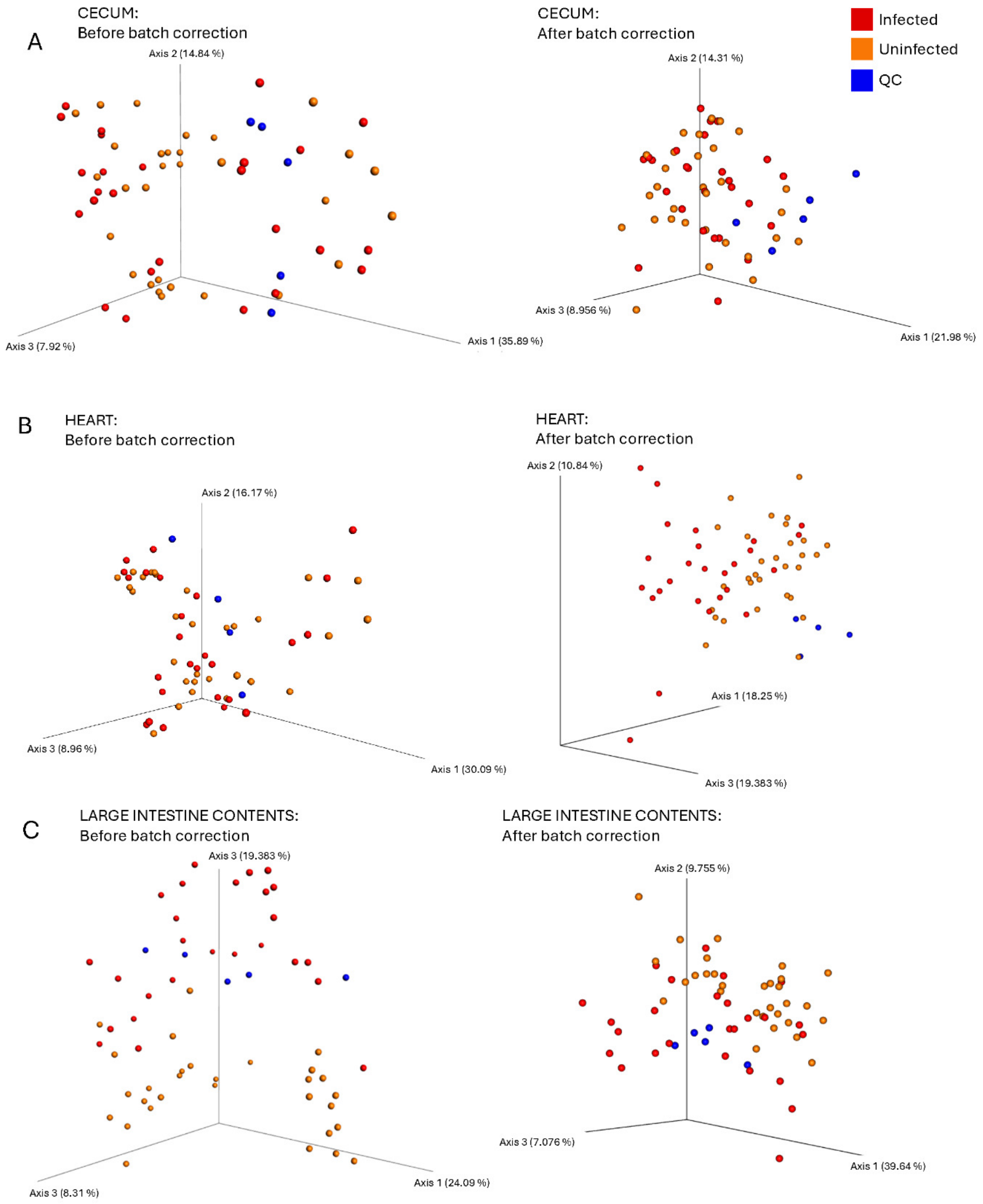

Fig. S6. PCoA plots of the cecum (A), heart (B) and large intestine contents (C) before and after batch effect correction.

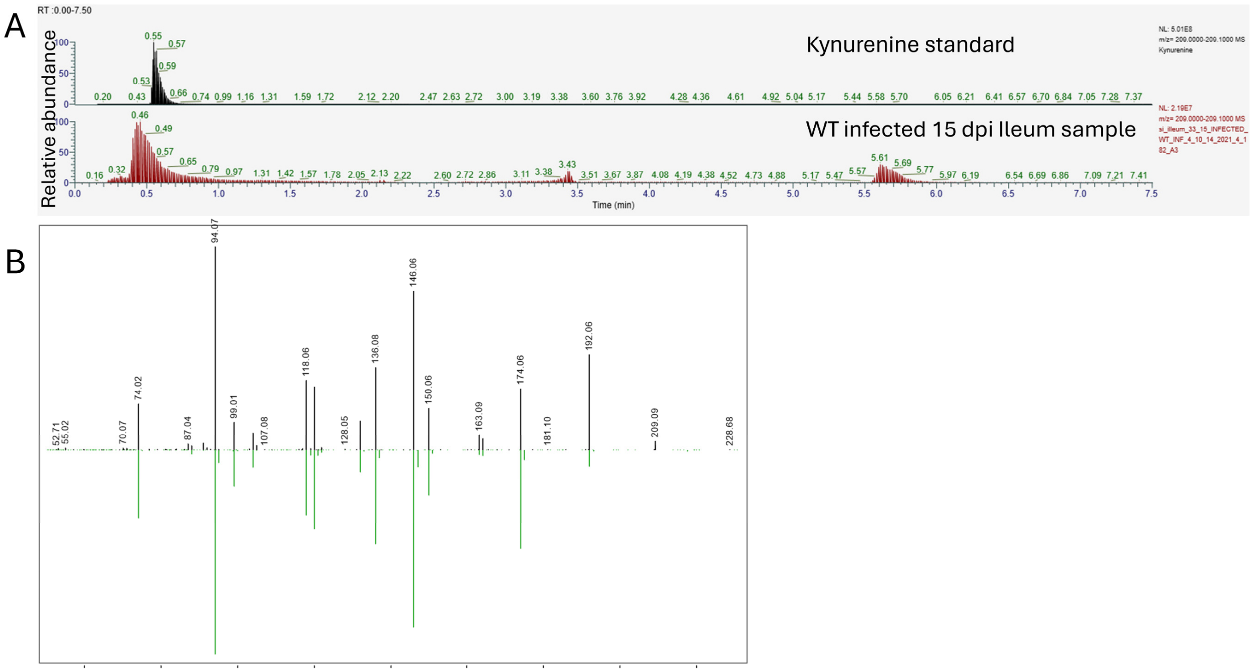

Fig. S7. Validation of kynurenine metabolite annotation. Extracted ion chromatograms of a pure kynurenine standard compared to annotated sample metabolites in WT acute infection ileum sample (A). Mirror plot of experimental MS/MS spectra (black) and GNPS reference MS/MS spectra for kynurenine (Green, B).
